# Revolutionizing Cow Welfare Monitoring: A Novel Top-View Perspective with Depth Camera-Based Lameness Classification

**DOI:** 10.3390/jimaging10030067

**Published:** 2024-03-08

**Authors:** San Chain Tun, Tsubasa Onizuka, Pyke Tin, Masaru Aikawa, Ikuo Kobayashi, Thi Thi Zin

**Affiliations:** 1 Graduate School of Engineering, University of Miyazaki, Miyazaki 889-2192, Japan; z321708@student.miyazaki-u.ac.jp (S.C.T.); hl18012@student.miyazaki-u.ac.jp (T.O.); pyketin11@gmail.com (P.T.); 2 Organization for Learning and Student Development, University of Miyazaki, Miyazaki 889-2192, Japan; aikawa@cc.miyazaki-u.ac.jp; 3 Sumiyoshi Livestock Science Station, Field Science Center, Faculty of Agriculture, University of Miyazaki, Miyzaki 889-2192, Japan; ikuokob@cc.miyazaki-u.ac.jp

**Keywords:** depth sensing camera, detection and tracking, lameness, random forest (RF), k-nearest neighbor (KNN), decision tree (DT)

## Abstract

This study innovates livestock health management, utilizing a top-view depth camera for accurate cow lameness detection, classification, and precise segmentation through integration with a 3D depth camera and deep learning, distinguishing it from 2D systems. It underscores the importance of early lameness detection in cattle and focuses on extracting depth data from the cow’s body, with a specific emphasis on the back region’s maximum value. Precise cow detection and tracking are achieved through the Detectron2 framework and Intersection Over Union (IOU) techniques. Across a three-day testing period, with observations conducted twice daily with varying cow populations (ranging from 56 to 64 cows per day), the study consistently achieves an impressive average detection accuracy of 99.94%. Tracking accuracy remains at 99.92% over the same observation period. Subsequently, the research extracts the cow’s depth region using binary mask images derived from detection results and original depth images. Feature extraction generates a feature vector based on maximum height measurements from the cow’s backbone area. This feature vector is utilized for classification, evaluating three classifiers: Random Forest (RF), K-Nearest Neighbor (KNN), and Decision Tree (DT). The study highlights the potential of top-view depth video cameras for accurate cow lameness detection and classification, with significant implications for livestock health management.

## 1. Introduction

Lameness in cows is a widespread and costly problem that has a detrimental impact on animal welfare and the dairy industry [1]. It manifests as abnormal gait and posture, resulting in pain, decreased productivity, reproductive issues, and increased mortality rates [2]. The early and accurate detection of cow lameness is crucial to promptly intervene and effectively treat the condition, mitigating its negative consequences [3]. The development of a computer vision-based cow lameness system holds tremendous potential in improving animal welfare and dairy farm economics [4]. Such a system can provide real-time monitoring of cow gait and behavior, facilitating the timely identification of lameness cases and enabling prompt intervention. By automating the detection process, the system reduces reliance on human observers, eliminates subjectivity, and enables the continuous monitoring of large herds. We propose an automated cow lameness detection system utilizing depth image analysis to streamline the process and minimize human surveillance. This system offers advantages such as reduced workload and the early prediction of lameness. Implementing this automated system improves animal welfare, optimizes farm management, and enhances cattle health and productivity, ultimately leading to increased profitability and sustainability in the dairy industry [5].

In our research, we focused on the detection of cow lameness using a depth camera. To achieve this, we employed the Detectron2 framework [6] for the simultaneous detection and segmentation of multiple cows. In our testing farm, the number of cows passing through during one period can range from a minimum of 56 to a maximum of 64. These periods occur both in the morning and evening. Given that 56 to 64 cows traverse the path between the milking station and the rest area twice a day, we utilize the Intersection over Union (IOU) technique for multi-cow tracking. A depth camera is strategically positioned along this pathway. For feature extraction, we calculate the highest points along the cow’s backbone, resulting in a feature vector with a length of 176 derived from a 132 × 176 depth image. To evaluate our approach, we experimented with various machine learning classifiers, including K-Nearest Neighbors (KNN), Random Forest (RF), and Decision Tree (DT). These classifiers were trained on a dataset that encompassed labeled instances of both healthy and lame cows.

## 2. Research Background and Related Works

Traditional methods for cow lameness detection, such as manual locomotion scoring, often suffer from limitations in terms of accuracy and the ability to promptly identify mild lameness. As a result, there is a growing demand for advanced technologies and automated systems that can improve the accuracy and timeliness of lameness detection and monitoring in cattle. Several approaches have been explored in the realm of cow lameness detection. Some methods involve the use of 2D videos and deep learning algorithms, such as convolutional neural networks (CNNs) [7] and Mask R-CNNs [8]. These approaches have been applied to extract features critical for assessing lameness, such as spine shape and leg distances [9,10]. Additionally, researchers have developed cow lameness prediction models based on sophisticated techniques like the You Only Look Once version 3 (YOLOv3) [11] and long short-term memory (LSTM) networks [12], achieving high accuracy in predicting lameness scores. Furthermore, there have been efforts to use cow back posture as a basis for classifying lameness in dairy cattle [13]. Incorporating sensor technology, some studies have explored the detection of lameness through locomotion or behavior analysis [14,15,16,17]. A neck-mounted mobile sensor system that combines local positioning and activity (acceleration) was tested and validated on a commercial UK dairy farm [18]. Cattle lameness causes considerable animal welfare problems and negatively affects the farm economy. Gait scoring techniques and claw health reports are commonly used for research and surveys, but few daily management solutions exist to monitor gait parameters from individual cows within a herd [19]. These sensor-based approaches provide valuable data for assessing cow health, but they also have their own set of challenges.

Recently, computer vision techniques, particularly depth image analysis, have emerged as promising alternatives for cow lameness detection. Depth image analysis harnesses the capabilities of depth-sensing cameras to extract precise features related to gait patterns and body posture [20,21]. Cattle behavior mainly refers to the animals’ continuous interaction with the environment and the way they express themselves. Hence, it is a valuable indicator in assessing the health and welfare of animals [22]. Utilizing cameras, depth sensors, and advanced algorithms, these techniques excel in discerning variations in posture, gait, and other visual indicators. The working principles involve capturing images or video footage of cows in specific areas, followed by applying image processing techniques like filtering, segmentation, and feature extraction. Extracted features, encompassing limb positions, body posture, and hoof movement, are then subjected to analysis by advanced machine learning algorithms, including convolutional neural networks (CNNs) [23]. Cow gait recordings were made during four consecutive night-time milking sessions on an Israeli dairy farm using a 3D camera. A live, on-the-spot-assessed 5-point locomotion score was the reference for the automatic lameness score evaluation. A dataset of 186 cows with four automatic lameness scores and four live locomotion score repetitions was used for testing three different classification methods [24]. The computer vision technique has been rapidly adopted in cow lameness detection research due to its noncontact characteristic and moderate price [25]. This non-contact monitoring method offers the advantage of early detection. However, challenges in this domain include the need for larger datasets, real-time processing algorithms, and practical integration into dairy farming operations.

To address these challenges, our research presents an innovative approach for 3D images. By utilizing depth-sensing cameras [26,27], the ability of sensing 3D space using single cameras has been a widely investigated topic in image processing and computer vision [28]. Monitoring the growth and body condition of cows is essential for the optimal management of modern dairy farms. However, monitoring is rarely performed on commercial farms. Modern technologies based on three-dimensional (3D) shape analysis could address this problem [29]. By utilizing advanced computer vision techniques, we aim to enhance the accuracy and reliability of cow lameness detection. Body cleanliness is considered an important indicator for evaluating cow welfare. At present, assessing the cleanliness of different cow body parts is considered as a subjective and labor-intensive task. Automatic body cleanliness scoring needs to start with body part segmentation [30]. Our method focuses on multi-cow detection and segmentation [31,32], as well as tracking using IOU analysis. Additionally, we extract feature vectors from depth images, specifically targeting the highest points along a cow’s backbone spine. These features serve as input for three different machine learning classifiers, enabling the classification of lameness. This holistic approach seeks to contribute to the field by offering a robust and efficient solution that can effectively handle cow lameness detection, addressing this critical issue in dairy farming operations.

## 3. Materials and Methods

**Figure 1:**
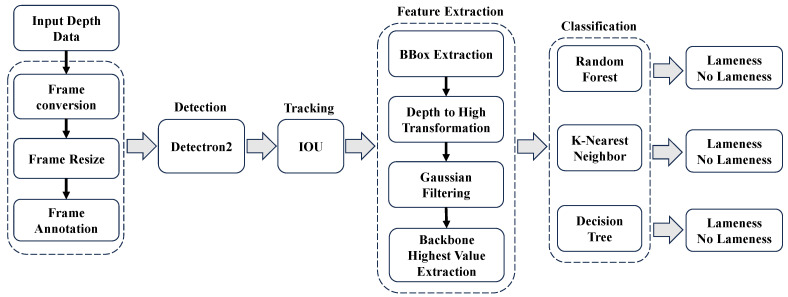
Flow diagram for proposed research.

Our proposed system aims to develop a robust and accurate cow lameness classification system by leveraging depth image analysis. The objective is to automatically identify and classify lameness in cows based on their movement patterns captured through depth imaging. This system offers a non-invasive and objective approach for early lameness detection, enabling timely intervention and improved animal welfare. The proposed system consists of five main components: data preparation, automatic cow detection, tracking, feature extraction, and classification. Figure 1 illustrates the research methodology we propose.

### 3.1. Data Collection and Preprocessing

**Figure 2:**
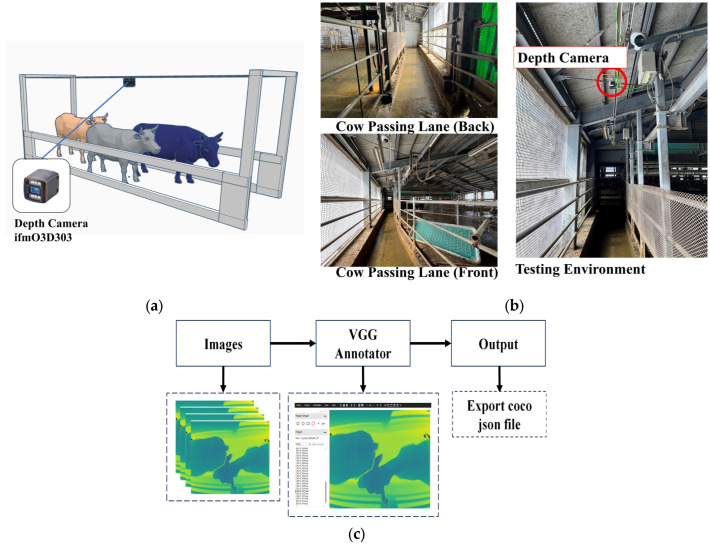
(**a**) Illustration of camera setting. (**b**) Testing environment. (**c**) Data preparation process.

The datasets used in this study were captured using a depth camera (ifm03D303) at the Kunneppu Demonstration Farm in Hokkaido Prefecture, Japan. The depth camera was strategically positioned at a height of 3 m from the ground to capture comprehensive information about cow movements. The camera was placed in the middle of the pathway between the entrance and exit gates. Furthermore, the indoor house featured a concrete floor, as illustrated in Figure 2a,b. This camera setting ensured an optimal view of the cows and enabled accurate depth measurements. To collect the data, the depth camera captured three-dimensional (3D) information about the cows’ movements. The distance data obtained by the camera were stored in CSV format, with each row representing a frame. The distance measurements were recorded for various points within the captured field of view. A VGG annotator was used to make the annotation of cow regions. Figure 2c shows the data preparation process.

In the preprocessing stage, the depth data captured by the camera are reshaped into an image size of 132 × 176 pixels. The research work utilized a dataset of 4944 depth data images, which were annotated for cow detection. Among them, 4120 were used for training the customized cow detection model. These training images contained a total of 4302 cow instances. For validation purposes, a subset of 824 images was selected, which included 915 cow instances. Currently, we employ a random training split of 80% and validation split of 20% from a total of 4944 frames. In the future, we plan to enhance the robustness of our model by incorporating validation data from different dates. In Table 1, detailed information about the dataset is presented.

### 3.2. Automatic Cow Detection

The proposed system employs the robust Detectron2 framework [33] for the purpose of customized cattle detection. This advanced framework harnesses the power of deep learning techniques to identify cows precisely and automatically within depth images. To achieve this level of accuracy, the system undergoes a fine-tuning process with specialized datasets containing cow-specific visual data. By adapting a pre-trained model to the distinctive visual characteristics of cows, the system significantly enhances its predictive accuracy, ensuring reliable and efficient cattle detection.

#### 3.2.1. Noise Removing

**Figure 3:**
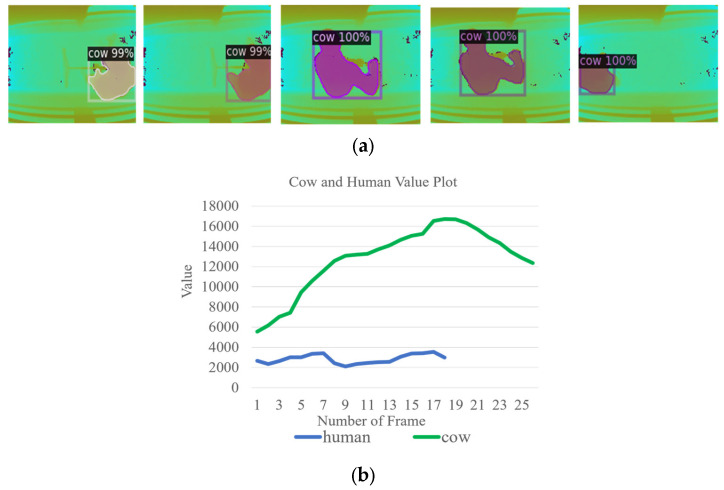
(**a**) Noise region (human). (**b**) The process of noise removal (human).

During the cow detection process, our proposed system operates continuously throughout the day, monitoring the movement of 56 to 64 cows between the milking production area and the resting area. This activity occurs during two time periods: in the morning from 5 a.m. to 8 a.m., and in the evening from 2 p.m. to 5 p.m. During these times, the farmer engages in pathway cleaning tasks. In Figure 3a, an example of our detection model identifying a human region as a cow is shown. This is considered a noise region, and it is necessary to eliminate this erroneous detection. To detect the cow regions accurately, a process is employed where the pixel values of the detected regions are summed and analyzed. This analysis aids in setting a pixel sum threshold that effectively distinguishes between cows and human areas, enabling the system to remove the human region. By effectively excluding these regions, the system focuses on the cow region, enabling the more reliable and precise analysis of cow lameness. Figure 3b presents the sum of detected cows and human regions. In our research, we establish the detected cow region as encompassing areas that exceed a defined threshold value (Th > 4000).

#### 3.2.2. Cow Depth Region Extraction

**Figure 4:**
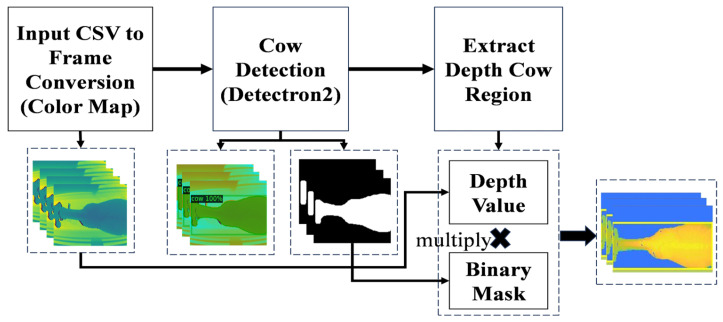
Cow depth region extraction.

After removing human noise region, we need to obtain the depth value of the detected cow region. To accomplish this, a binary mask specific to the cow-detected areas must be generated using our detection model. This binary mask is then applied to the original depth images through a multiplication process. After this process, we obtain the depth value for the cow region. Figure 4 presents the cow depth region extraction from detection.

### 3.3. Automatic Cow Tracking

**Figure 5:**
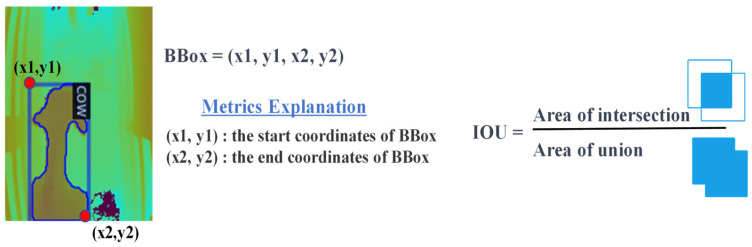
Cow tracking with IOU.

For tracking, our system relies on the Intersection over Union (IOU) metric to assess the overlap of bounding boxes across consecutive frames. By analyzing IOU values and adjusting coordinates according to a predefined threshold, the system proficiently tracks the movement of cows. Figure 5 provides a visual representation of the IOU tracking process, showcasing the comparison between IOU values in the current frame and the previous frame with a designated IOU threshold.

**Figure 6:**
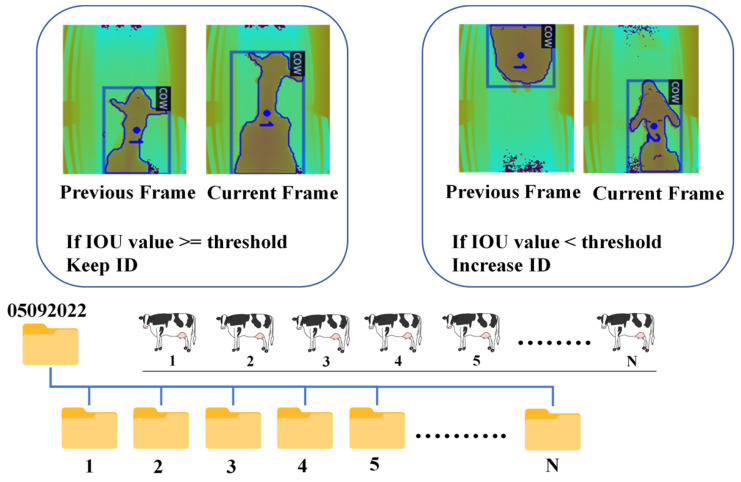
Cow tracking and saving to folder according to tracking IDs.

When the IOU value between bounding boxes in consecutive frames exceeds or equals the specified threshold, the system retains the same tracking ID. Conversely, if the IOU value falls below the threshold, a new tracking ID is assigned. Following this tracking process, the system efficiently organizes and archives the tracked cows, saving them into individual folders corresponding to their respective track IDs, which are sequentially numbered as 1, 2, 3, and so on. This structured approach streamlines data management and facilitates easy access to and analysis of the tracked cow data within their designated folders. Figure 6 illustrates the process of cow tracking and saving to folders according to tracking IDs.

### 3.4. Cow Lameness Classification

The cow lameness classification system consists of two main components: feature extraction and classification. The feature extraction component analyzes sensor data to extract relevant information related to cow lameness, while the classification component uses three machine learning algorithms to classify the extracted features into different lameness categories. This system aims to enhance the early detection and monitoring of cow lameness, ultimately improving the welfare of cattle.

#### 3.4.1. Cow Lameness Classification

**Figure 7:**
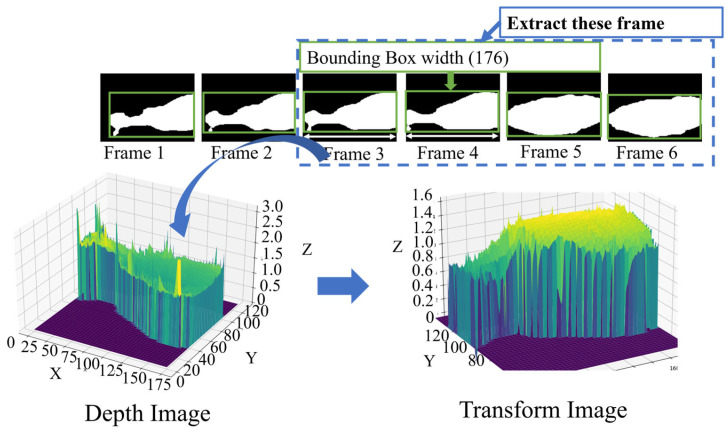
Depth to high transformation.

To perform feature extraction on a cow frame with specific criteria, such as extracting frames where the bounding box width is full size (176), transforming depth values, applying a Gaussian filter, and finding the maximum value of the cow backbone. The process of extracting features from cow frames involves several steps. Firstly, frames with a desired bounding box width of 176 are selected. Next, the depth values in these frames are transformed using Equation (1), enhancing the representation of cow depth. Figure 7 presents the illustration of depth to high transformation.

(1) $$transform=distance-depth_{img\left(x,y\right)}$$

**Figure 8:**
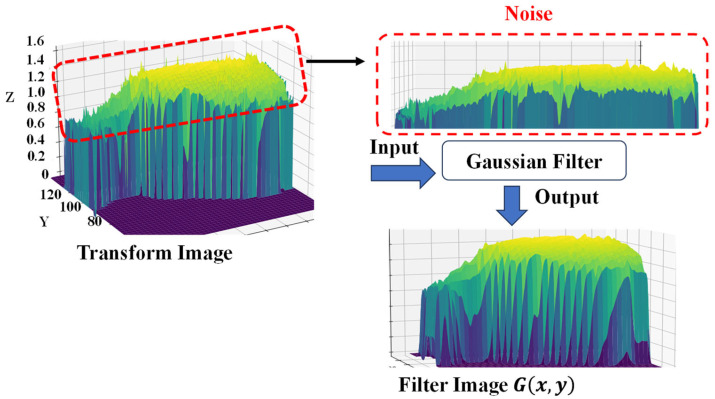
Gaussian filter for noise reduction.

To further process the transformed values, a Gaussian filter is applied, which reduces noise and smooths the data. This is achieved by convolving the transformed values with a Gaussian function calculated using Equation (2). Figure 8 presents the illustration of the filter image.

(2) $$\;G\left(x,y\right)=\frac{1}{2\pi \sigma^{2}}e^{-\frac{x^{2}+y^{2}}{2\sigma^{2}}}$$

**Figure 9:**
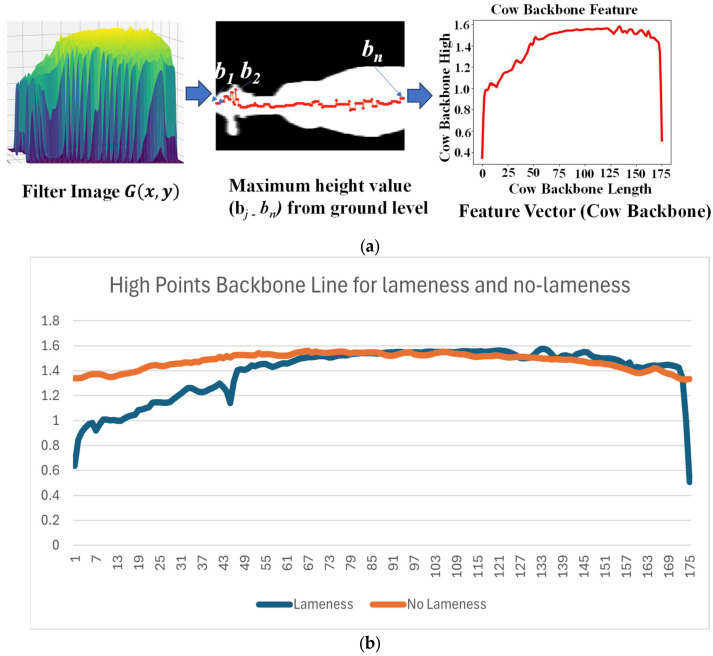
(**a**) Extraction of maximum backbone value. (**b**) Maximum highest points for backbone.

Following the process of high transformation and Gaussian filtering, our process culminates in the extraction of the highest points along the cow’s backbone line. This extraction is performed using Equation (3). Subsequently, these extracted highest points are harnessed as feature vectors, encapsulating essential characteristics pivotal for subsequent analysis and classification. Figure 9a provides an illustrative depiction of how the highest backbone values are extracted.

(3) $$backbone\left[j\right]=\max\left(G\left(i,j\right)\right)\;\;for\;i=1,2,\ldots ,m,\;\;1\leq j\leq n$$

where transform: transformed value from equation; distance: camera distance from ground to 2.8 m above; $depth_{img\left(x,y\right)}$: depth image value at coordinates $\left(x,y\right)$; $G\left(x,y\right)$: the value of Gaussian function at coordinates $\left(x,y\right)$; σ(sigma): the standard deviation of the Gaussian distribution; m: the number of rows in ‘G’, which in this case is 132; n: the number of columns in ‘G’, which in this case is 176.

#### Lameness and No-Lameness Cows

In Figure 9b, we can observe that most of the lame cows exhibit a curved backbone, measured from the starting point (their head and neck), mostly with values lower than 1.2. In contrast, for non-lame cows, their highest point in the backbone is straight, predominantly with values greater than 1.2.

The resulting values are then analyzed, and the maximum value in the region of interest corresponds to a prominent feature of the cow’s backbone. After extracting the highest values along the cow’s backbone as feature vectors, our next step involves their classification using methods such as K-Nearest Neighbor (KNN), Random Forest, and Decision Tree.

## 4. Performance Evaluation

The performance evaluation section consists of three parts: detection accuracy, tracking accuracy, and classification accuracy.

### 4.1. Automatic Cow Detection Accuracy

To evaluate the detection accuracy of our system, we collected testing data over a period of three days, encompassing both morning and evening sessions. Specifically, on 3 September (whole day) and 4 September (morning), a total of 56 cows were included in the dataset. For the evening of 4 September and the whole day of 5 September, we expanded the dataset to include 64 cows. These dates were intentionally chosen because during this period, accurate ground truth lameness scores were available from experts at the cow farm. Notably, our system successfully detected all cows during the entire duration of the three days, in both the morning and evening sessions. Remarkably, our system achieved an impressive average detection accuracy of 99.94%, demonstrating its high performance and reliability in accurately identifying and tracking cows. The evaluation results for automatic cow detection are presented in Table 2.

(4) $$Accuracy\;of\;Cow\;Detection=\frac{TP+TN}{TP+FP+TN+FN}$$

where TP: True Positive; FP: false positive; TN: True Negative; FN: False Negative.

### 4.2. Automatic Cow Tracking Accuracy

For evaluating the performance of cow multi-object tracking, we have adopted the Multi-Object Tracking Accuracy (MOTA) metric [34]. The MOTA calculation is defined by Equation (5). The evaluation results for automatic cow tracking are presented in Table 3. The average accuracy was computed for all testing dates, yielding an overall accuracy of 99.92% over a three-day testing period.

(5) $$MOTA=1-\frac{\sum_{t}\left(FN_{t}+FP_{t}+{IDS}_{t}\right)}{\sum_{t}GT_{t}}\;$$

where IDS: ID Switch, GT: ground truth, FN: Missed Tracks, FP: False Tracks.

### 4.3. Cow Lameness Classification Accuracy

In this classification task, we classed 45 cows as No Lameness and 31 cows as Lameness. For the training, we used 885 frames for No Lameness and 491 frames for Lameness. For the testing, we used 229 frames for No Lameness and 116 frames for Lameness. The RF model achieved an accuracy of 82.3% during training, the KNN model achieved 81.2%, and the DT model achieved 70.4%. For the testing phase, the accuracy results were 81.1% for RF, 78.2% for KNN, and 69.2% for DT. The evaluation results for lameness classification are presented in Table 4.

**Figure 10:**
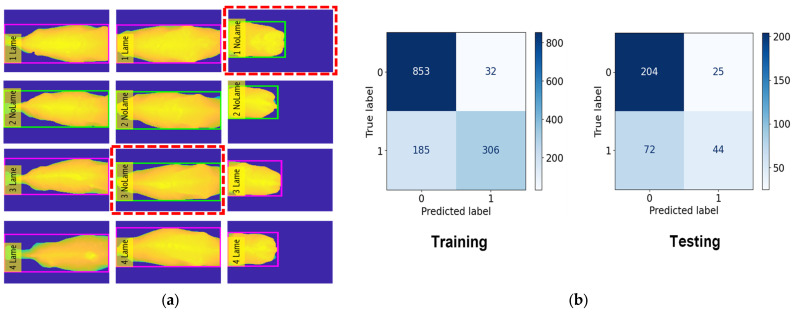
(**a**) Lameness Testing Results with RF and (**b**) Confusion Matrix with RF.

**Figure 11:**
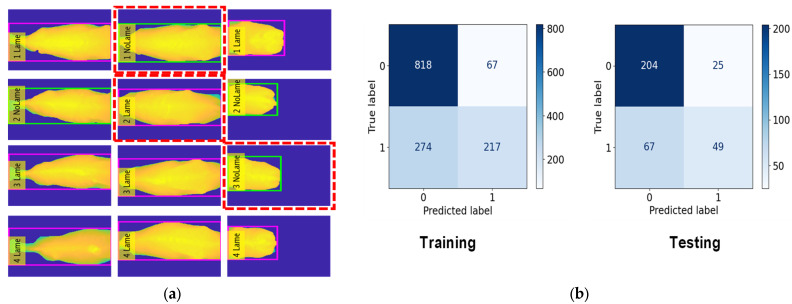
(**a**) Lameness Testing Results with KNN and (**b**) Confusion Matrix with KNN.

**Figure 12:**
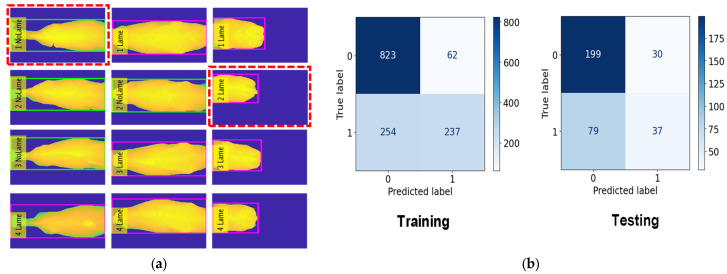
(**a**) Lameness Testing Results with DT and (**b**) Confusion Matrix with DT.

Figure 10, Figure 11 and Figure 12 provide a comprehensive visual representation of our classification results using different algorithms. In Figure 10, we present the Lameness Testing Results obtained with the Random Forest (RF) classifier in Figure 10a, while Figure 10b displays the associated Confusion Matrix. Moving on to Figure 11a, it showcases the Lameness Testing Results achieved with the K-Nearest Neighbor (KNN) algorithm, and Figure 10b offers insights into the corresponding Confusion Matrix. Lastly, Figure 12a illustrates the Lameness Testing Results derived from the Decision Tree (DT) classifier, and Figure 10b elucidates the Confusion Matrix pertaining to DT. These figures collectively offer a visual perspective on the effectiveness of each classification method in distinguishing between ‘Lame’ and ‘Not Lame’ cow conditions. In Figure 10, Figure 11 and Figure 12a, we conducted a detailed analysis of the system’s classification results. In these figures, we present the outcomes of our classifiers, with red dotted lines denoting instances of incorrect predictions, while all other points represent correctly classified frames. This visual representation helps us to discern the accuracy and precision of our classification models, providing valuable insights into their performance and their ability to distinguish between ‘Lame’ and ‘Not Lame’ cow conditions.

## 5. Discussion

In this section, we delve into the details and outcomes of our proposed computer vision system, emphasizing its capabilities in cow automatic detection, depth region extraction, and automatic tracking, particularly in a real-world scenario where cows share their path with farmers. We also discuss the challenges related to human detection and the system’s performance in cow lameness classification. Furthermore, we outline the limitations of our current approach and articulate our plans for future enhancements.

Our computer vision system was rigorously tested in a practical environment over three days, involving the monitoring of a fluctuating population of cows ranging from 56 to 64. The system’s effectiveness in automatic cow detection, depth region extraction, and tracking was assessed, and the results are presented in Table 2 and Table 3, which illustrate the system’s testing accuracy in cow detection and tracking.

One of the notable challenges encountered in this real-world setting is the presence of humans, particularly farmers, in the same passage as the cows. Humans can easily be misidentified as cows, leading to false detections. However, our system incorporates a detection thresholding mechanism, which helps distinguish cows from humans, thereby reducing false positives. This feature contributes to the reliability of our system’s cow detection and tracking capabilities.

Beyond cow detection and tracking, our system addresses the critical issue of cow lameness classification. Lameness in cows is a key indicator of their health and well-being, and timely identification can lead to improved animal welfare. To classify cow lameness, we utilized the highest backbone values of cows as feature vectors and employed three different machine learning algorithms.

However, as shown in Figure 10, Figure 11 and Figure 12, our classification system does exhibit limitations. These limitations stem from our reliance on a subset of features—the highest backbone values—for classification. Consequently, there are instances where our system produces incorrect predictions. To overcome the limitations of our current approach and further enhance the system’s capabilities, we will focus on feature extraction methods: histograms of depth, depth gradients, depths based on landmasks. Using these approach methods will gain better accuracy and better performance.

In the future, we plan to compare the cow lameness classification results with the population frequency in an average dairy herd in Japan. Our testing classification result achieved 81.1% for our farm. Our testing farm comprises over 100 cows. Human monitoring for all cows incurs substantial costs and yields inaccurate results. However, our system, utilizing only one depth camera, not only saves significant costs but also provides accurate results. We recognize the potential impact of uneven floor surfaces on our testing accuracy, particularly in the context of focusing on cows’ highest points within indoor settings with concrete flooring. We plan to incorporate adjustments in our methodology to account for the uneven floor surface as a contributing factor to any decrease in accuracy. Additionally, we will consider the age of cows in our future studies and commit to integrating this consideration into our research methods.

## 6. Conclusions

In this study, our proposed system was subjected to rigorous real-world testing, involving a substantial cohort of 56 to 64 cows. Observations were conducted twice daily, encompassing both morning and evening sessions. The aim was to assess the system’s practical applicability and resilience in the realm of cow lameness detection and classification. Our approach involved harnessing the precise characteristics of the cow’s backbone spine line as a feature vector, coupled with the utilization of machine learning algorithms. The remarkable outcomes achieved underscore the effectiveness of this approach in automating the categorization of cow lameness levels. The consistency of our testing across varying times of the day and diverse cow behaviors provided invaluable insights into the system’s reliability and robustness under real-world conditions. This extensive validation further highlights the potential for the seamless integration of the proposed system into contemporary livestock management practices.

In conclusion, our study marks a significant stride forward in the quest for automated cow lameness detection and classification. The fusion of meticulous real-world testing involving 56 to 64 cows, combined with strategic feature selection and machine learning algorithms, underscores the practical viability of our system. This advancement paves the way for improved animal welfare and more efficient farm operations, setting the stage for the adoption of our technology in real-world agricultural settings. As we venture forward, refining and expanding upon these findings will undoubtedly contribute to the ongoing progress in precision livestock monitoring.

## Figures and Tables

**Table 1:** Dataset information.

Dataset	Date	Time	#Frames	#Instances
Training	22 January 2023 (Morning)	05:00–08:00	4120	4302
Validation	22 January 2023 (Morning)	05:00–08:00	824	915

**Table 2:** Automatic cow detection accuracy.

Date	Time	#Cow	TP	TN	FP	FN	Accuracy (%)
3 September 2022	AM	56	1217	0	0	0	100
	PM	56	1273	0	4	0	99.69
4 September 2022	AM	56	1240	0	0	0	100
	PM	64	1836	1	4	0	99.95
5 September 2022	AM	64	1736	0	0	0	100
	PM	64	1477	0	0	0	100
Average Accuracy							99.94

**Table 3:** Automatic cow tracking accuracy.

Date	Time	#Cow	GT	FP	FN	IDS	MOTA (%)
3 September 2022	AM	56	1247	0	0	0	100
	PM	56	1297	0	2	3	99.61
4 September 2022	AM	56	1257	0	0	0	100
	PM	64	1843	1	0	1	99.89
5 September 2022	AM	64	1778	0	0	0	100
	PM	64	1498	0	0	0	100
Average Accuracy							99.92

**Table 4:** Performance matrix for training and testing accuracy.

Dataset	Date	Period	Classification Accuracy		
			RF (%)	KNN (%)	DT (%)
Training	3 September 2022, 4 September 2022, 5 September 2022	a.m., p.m. a.m., p.m. a.m.	82.3	81.2	70.4
Testing	5 September 2022	p.m.	81.1	78.2	69.2

## Data Availability

The datasets featured in this study are available upon request from the corresponding author.
